# A Review of Pedestrian Indoor Positioning Systems for Mass Market Applications

**DOI:** 10.3390/s17081927

**Published:** 2017-08-22

**Authors:** Alejandro Correa, Marc Barcelo, Antoni Morell, Jose Lopez Vicario

**Affiliations:** Telecommunications and Systems Engineering Department, Universitat Autònoma de Barcelona, Bellaterra 08193, Spain; marc.barcelo@uab.cat (M.B.); antoni.morell@uab.cat (A.M.); jose.vicario@uab.cat (J.L.V.)

**Keywords:** indoor localization, pedestrian tracking, navigation, indoor location based services

## Abstract

In the last decade, the interest in Indoor Location Based Services (ILBS) has increased stimulating the development of Indoor Positioning Systems (IPS). In particular, ILBS look for positioning systems that can be applied anywhere in the world for millions of users, that is, there is a need for developing IPS for mass market applications. Those systems must provide accurate position estimations with minimum infrastructure cost and easy scalability to different environments. This survey overviews the current state of the art of IPSs and classifies them in terms of the infrastructure and methodology employed. Finally, each group is reviewed analysing its advantages and disadvantages and its applicability to mass market applications.

## 1. Introduction

The estimation of the position of a target in an outdoor environment is usually solved employing Global Navigation Satellite Systems (GNSS). Notwithstanding, in the case of indoor environments there is an absence of a standard positioning system that can be worldwide applied. For this reason, the research efforts of authors working in the field of positioning have been recently focused in indoor scenarios. Indoor Positioning Systems (IPS) have been designed for providing information about the position of a person or object inside a building. In fact, the evolution of IPS facilitates the creation of Indoor Location Based Services (ILBS) which build applications on top of the knowledge of the position. Examples of these kinds of services are the location of products stored in a warehouse, the tracking of equipment inside a hospital, the guidance of firemen inside buildings with reduced visibility due to smoke, among others like the guidance of people inside airports or the development of assisted living systems for elderly care. Indeed, the predicted market value of indoor location services for 2020 is USD 10 billion [1]. Therefore, there is a special interest in developing IPS that can be easily scaled to mass market applications and deployed in millions of buildings in the world. There are three main requirements for an IPS that aims to be applied to mass market applications: (i) the system must provide accurate position estimations; (ii) the system must be easily scalable; and (iii) the cost of the system infrastructure should be reduced.

The current trend to reduce the cost of the systems is to use the wireless infrastructures already deployed for communications as landmarks for positioning. Among the multitude of available technologies for communications such us LTE, WiFi, Bluetooth, Wireless Sensor Networks (WSN) or Ultra Wide Band (UWB), the WiFi technology is the most commonly employed because it is already worldwide deployed. Although WSNs are also commonly used due to its key role in the Internet of the Things (IoT) and the future of smart cities. Similarly, the development of the Microelectromechanical Systems (MEMS) provide us with low cost inertial sensors that can also estimate the position of a pedestrian without the need of any infrastructure in the building. Note that most of these technologies are already available in nowadays smartphones, converting the smartphone in the perfect device for mass market positioning systems.

Numerous studies have attempted to review the current state of art of IPS. Since the first review in 2001 by Hightower et al. [2], many new reviews have appeared. For example in 2002, Pahlavan et al. [3] presented a review of the state of the art focusing on systems for indoor environments. More recent revisions are presented in [4,5] covering hybrid systems of network based technologies. Similarly, inertial based systems are reviewed in [6]. There are other surveys that cover specific kinds of systems, like fingerprinting [7,8], or a specific technology, like UWB [9]. Other authors reviewed the state of the art from a bayesian estimation perspective as in [10] or in [11].

Despite the numerous surveys in IPS, none of them performed a comprehensive analysis of the state of the art of IPS focusing on its application to the mass market. In this work we overview the state of the art of IPS analysing the advantages and disadvantages of the reviewed systems and its applicability to mass market applications. First of all, we classify and review individual IPS and then we review the most promising hybrid methods for mass market applications.

Traditionally IPS systems can be classified into three groups:

- **Network based systems:** these systems are build on the top of a wireless network deployed in the scenario and use the information of the wireless signals to estimate the position of the user carrying a wireless device.
- **Inertial based systems:** these systems use self-contained sensors that measure the motion of the user and estimate its position relative to the starting point without the need of any physical infrastructure deployed in the building.
- **Hybrid systems:** these systems jointly combine two or more different methods in order to enhance the estimation of position.

**Figure 1:**
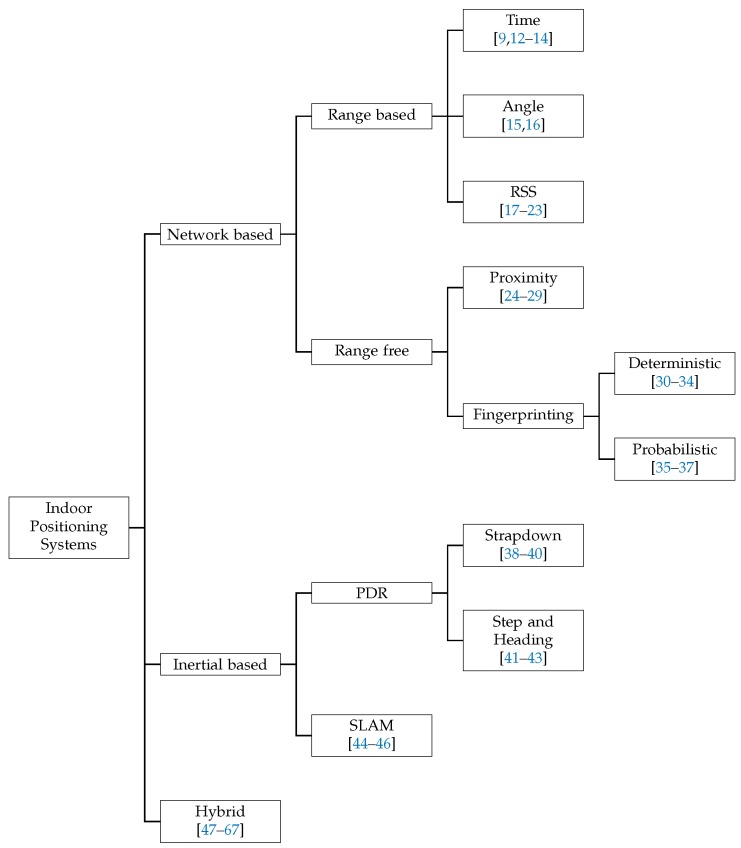
Classification of Indoor Positioning Systems. Time [9,12,13,14], Angle [15,16], RSS [17,18,19,20,21,22,23], Proximity [24,25,26,27,28,29], Deterministic [30,31,32,33,34], Probabilistic [35,36,37], Strapdown [38,39,40], Step and Heading [41,42,43] , SLAM [44,45,46], Hybrid [47,48,49,50,51,52,53,54,55,56,57,58,59,60,61,62,63,64,65,66,67].

A complete classification of IPS is shown in Figure 1 including references to remarkable works. Note that hybrid systems are not subclassified. There are lots of possible combinations of IPS that can form an hybrid system and a general classification of these systems is not feasible. In this work we only focus on those hybrid systems that can be scaled to mass market applications.

The rest of the paper is organized as follows: Section 2 reviews the network based IPS. Section 3 is focused on the review of inertial based systems whereas Section 4 reviews the state of the art of hybrid systems for mass market applications. Finally, in Section 5 we present the conclusions and some possible future lines of work.

## 2. Network Based Systems

There are many different wireless networks that can be deployed in an indoor environment. From the typical WiFi networks that are deployed in millions of buildings around the world for providing internet access, to the WSNs designed for the IoT or the popular Bluetooth beacons among other alternatives such as the UWB networks. Leaving aside the election of the wireless network, which obviously will determine the accuracy and precision of the IPS, we can classify the network based IPS systems according to the information obtained from the wireless signals into two groups: (i) range based methods and (ii) range free methods.

Range based methods extract geometric information (distance or angle) from the signals of different anchor nodes in the wireless network and then combine the geometric constraints of each anchor to obtain the position of the user. Alternatively, the range free methods are based on the inter-node connectivity information or in the identification of signal features patterns that are location dependent.

### 2.1. Range Based

There are different ways for extracting geometric information from the wireless signals, the most common ones are the methods based on the propagation time of the signal, between the transmitter and the receiver, the Angle of Arrival (AoA) or the Received Signal Strength (RSS). In the following, we briefly detail the fundamentals of each class of methods analyzing its advantages and disadvantages.

#### 2.1.1. Time

**Figure 2:**
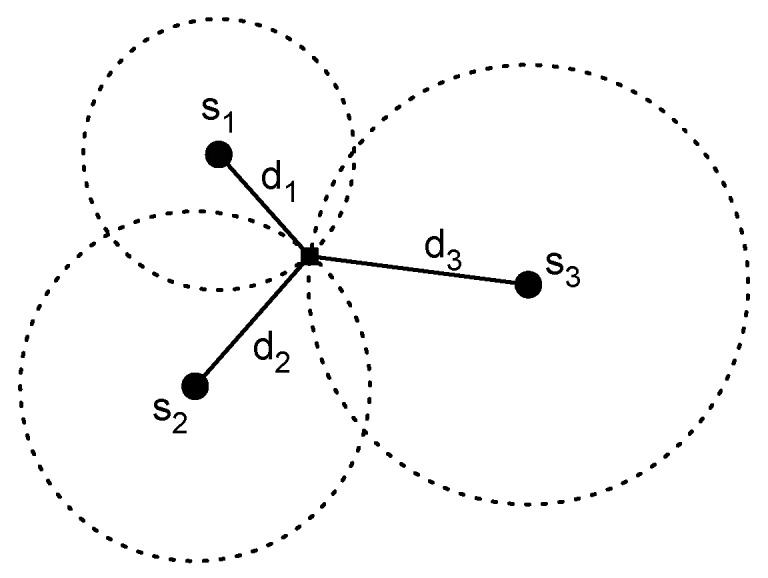
Lateration method concept.

Time based localization algorithms measure the propagation time of a signal between the transmitter and the receiver, also known as Time of Flight (ToF) and compute the distance between the user and the anchor node *d* as follows,

(1) d=Δtv,

where Δt is the ToF and *v* is the propagation speed. The simplest approach is known as Time of Arrival (ToA). In this case the transmitter includes in the radio packet the time when the signal is transmitted and the receiver computes the reception time. Thus, the receiver has all the information for computing the distance. The position of the user can be obtained employing a lateration method, if the distance to multiple anchors nodes is known. The idea behind lateration methods (see Figure 2) is to estimate the position as the point of intersection of three circles. These circles have the center situated in the position of the anchor nodes and the radius is equivalent to the estimation of distance computed. Note that three different circles are necessary in order to obtain a position estimation in a two dimensional space.

It is fundamental in a ToA method to take into account that ToA methods require synchronization between all nodes, as the time of reference must be the same in all cases. This can be a problem for certain kind of wireless networks with simple low power devices and high restrictions in the algorithm complexity, as it is the case of WSNs. An alternative method that relaxes the synchronization constraint is the Time Difference of Arrival (TDoA). There are two main implementations of TDoA:

- The first TDoA method computes the difference in the ToA of a signal transmitted to two different receivers. For each TDoA measurement the transmitter must be in a hyperboloid with a constant range difference between the two receiver positions [4]. This method relaxes the synchronization constraint to the receivers.
- The second TDoA method is based on the difference in the ToA of two different signals with different propagation times. Usually, the first signal is the radio packet and the second one is a kind of sound signal due to the difference in the propagation speed between the electromagnetic waves (propagate at the speed of light ≈300,000 km/s) and the acoustic waves (propagation speed ≈340 m/s) [68]. This method does not need synchronization but the nodes must include additional hardware in order to send two kind of signals simultaneously.

One example of a time based positioning system is the Active Bat system [12]. This system is based on the TDoA of ultrasound signals. The user carries a transmitter and the signals are received by a grid of ceiling mounted receivers, which are synchronized using a wired connection. The system reports accuracies within 9cm for the 95% of measurements. The main disadvantages of the system are related to the placement of the receivers in the ceiling which increases the cost and reduces the scalability.

Another example based also in ultrasound signals is the Cricket system [13]. The working principle of the Cricket system is similar to the Active Bat system but in this case the computation of the position is performed by the user which carries an ultrasound receiver. A set of ultrasound transmitters are deployed around the building, which also transmit radio frequency signals for synchronization.

More recent works are based on the UWB technology, which improves the ranging accuracy due to the large bandwidth used [14]. The use of a large bandwidth allows to implement shorter pulses which increase the time resolution and accuracy of the ToF estimations. Therefore the accuracy of the positioning system is also improved. The fundamental limits of wide band localization methods are determined in [69,70] where the problem is extended to cooperative networks. More information about UWB systems can be found in [9,14]. The main problem of UWB technology when applied to mass market applications is the level of deployment of these networks around the world. Unlike WiFi, UWB systems are not worldwide deployed and thus any IPS based on UWB will have additional expenses due to the infrastructure cost. Nevertheless, this problem will be circumvented with the deployment of the Fifth Generation (5G) of cellular networks around the world, which will also employ signals with large bandwidth and centimeter level ranging accuracy [71].

Time based localization methods are susceptible to errors produced for inaccuracies in the clocks or errors in the time estimation. Take into account that for a signal traveling at light speed 1μs of error corresponds to an approximate distance error of 300m. Furthermore, Non Line of Sight (NLOS) conditions produce a positive bias in the distance estimation. Therefore time based methods must include methods for detecting NLOS conditions increasing the complexity of the algorithms.

In general, time based systems obtain highly accurate position estimations and can be scaled to large areas by adding new nodes to the network. The scalability to multiple users is limited by the possible collisions when multiple users coexist in the same area. Thus, collision avoidance mechanism must be implemented increasing the overall complexity of the algorithm. Finally, the cost of the system is determined by the network technology and the number of nodes required.

#### 2.1.2. Angle

Angle based localization methods use the angle of arrival of a signal to compute the position of the receiver. The working principle is similar to time based methods but instead of using the distances to the anchor nodes the angles are used. There are typically two main methods of obtaining the AoA of a signal [17]:

- Use an array of sensors (for ultrasound systems) whose locations relative to the node center are known and use the difference in the ToA of the signal at each sensor to compute the AoA of the anchor node. In the case of using radio signals the array of sensors is replaced by an antenna array.
- Use two or more directional antennas pointing to different directions and with overlapping main beams. Then compute the AoA as a function of the ratio of the RSS of the individual antennas.

**Figure 3:**
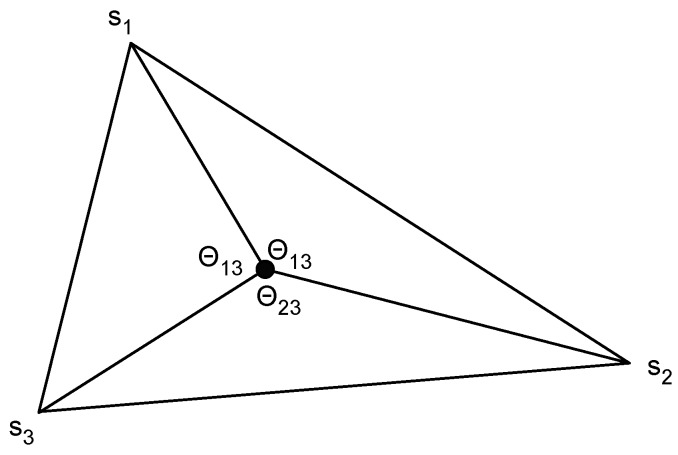
Triangulation method concept.

Once the AoA of multiple anchor nodes is estimated, the computation of the position is done using triangulation. The basic idea is shown in Figure 3. If the position of the vertices of a triangle are known, it is possible to compute the position of any node inside the triangle knowing the angle at which the interior point sees the vertices [15]. There are many different ways of solving the triangulation problem, in [16] the most common methods are reviewed and a new method that does not take into account the ordering of the anchor nodes is presented.

The main disadvantage of AoA based methods is the increase in the cost of the system due to the additional hardware, as these systems need arrays of sensors or antennas. Furthermore, the computation of accurate angle estimations is expensive in terms of computational cost and is negatively affected by low Signal to Noise Ratio (SNR) and small errors in the estimations of the RSS or the ToA [72]. Therefore, the scalability of the system is limited by the increase of the cost. In general, AoA systems are not commonly employed for indoor localization due to the additional hardware and computational power required.

#### 2.1.3. RSS

**Figure 4:**
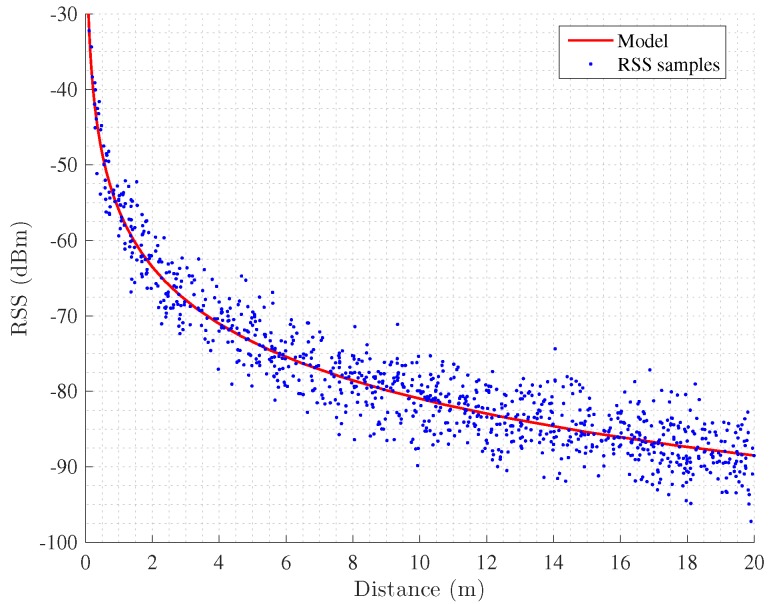
Calibration of a propagation model.

RSS based localization methods estimate the distance between the user and an anchor node using the received signal strength. These methods are based on the concept that the attenuation suffered by a signal travelling from a transmitter to a receiver depends on the distance travelled. In order to estimate the distance it is necessary to model the wireless environment using a propagation model. Traditionally, the log-distance path loss model is employed, where it is considered that the attenuation (in dB) is proportional to the logarithm of the distance travelled [17], that is,

(2) $$RSS=P_{1m}-10\alpha \log_{10}d-\gamma ,$$

where *d* is the distance between the receiver and the transmitter, $P_{1m}$ is a reference power measured in dBm at a distance of 1 m from the transmitter , α is the path loss exponent and $\gamma ∼N\left(0,\sigma_{\gamma }^{2}\right)$ models the effects produced by the shadowing. Note that in order to obtain the parameters of the model, i.e., $P_{1m}$ and α, a small calibration campaign is done at each scenario. The calibration process consists in the collection of the RSS in predefined positions with known distance to anchors and the computation of the model parameters, which is usually done using regression methods. Figure 4 shows the calibration process with the collected samples and the computed log-distance path loss model.

Once calibrated, distance is estimated according to the path loss model using the Maximum Likelihood Estimator (MLE), which for the case of the distance estimation is [18]:

(3) $$\hat{d}=10^{\frac{RSS-P_{1m}}{10\alpha }}.$$

As in the case of time based localization algorithms, the position of the user is estimated combining the distance information of multiple anchor nodes using a lateration method [19]. RSS based methods are attractive due to its inherent simplicity, as far as the RSS measurements are natively supported by most transceivers. Unfortunately, the variability of the wireless channel jointly with the attenuation of the signal due to walls, objects or the human body introduce errors in the distance estimation and makes the RSS based localization algorithms less accurate than time or angle based algorithms. A review of the main sources of error of RSS based algorithms can be found in [20] where the authors also include a list of recommendations for the appropriate implementation of RSS based algorithms.

An example of a RSS based localization system can be found in [19], where authors use the correlation between the RSS samples in nearby locations to fit different path loss models depending on the position of the user and therefore adapt to changes of the propagation model between areas of the same building. In [18] a cooperative method for the localization of the nodes in a WSN is presented. Other authors employ multiple receivers to enhance the ranging accuracy of RSS measurements and to provide coarse estimations of the heading of the user [22]. In [23] a Bluetooth based system is presented employing a stigmergic approach to mitigate the multipath fading. A comparison of the accuracy of the RSS based methods versus the time based methods is presented in [21] where the authors compute the Cramér-Rao bound under Gaussian and log-normal models.

RSS based systems can be easily scaled to large areas and multiple number of users due to the simplicity of the RSS measurements (note that the RSS measurements can be obtained even without having to be part of the network). Furthermore, the cost of the system infrastructure is minimum if WiFi networks are employed as they are already worldwide deployed. Unfortunately, the accuracy provided by RSS based systems is low and is negatively affected by the distance between the transmitter and the receiver.

### 2.2. Range Free

Range free methods are based on the connectivity information of a wireless network, which can be used to estimate the position without computing any range measurement to an anchor node. There are mainly two kind of range free algorithms:

- **Proximity methods:** these methods use the connectivity information to infer directly the position of the user based on the number of anchors in the neighbourhood.
- **Fingerprinting methods:** these methods are based on location dependent characteristics of the signals received from the wireless network. In a first step, a database of the characteristics and the real location where they were measured is collected. Then, in a second step, the position is estimated by selecting the position of the database sample that best matches the real data.

#### 2.2.1. Proximity

The proximity algorithms are based on the following simple idea: if a user is receiving a signal from an anchor node, the position of the user must be near the position of the anchor node. The operation mode is as follows: first, the user scans the channel looking for the radio signals from the anchors nodes. Once an anchor node is detected, the position of the user is estimated as the position of the anchor node. In the case of detecting more than one radio signal, the anchor node with the strongest received signal is selected.

**Figure 5:**
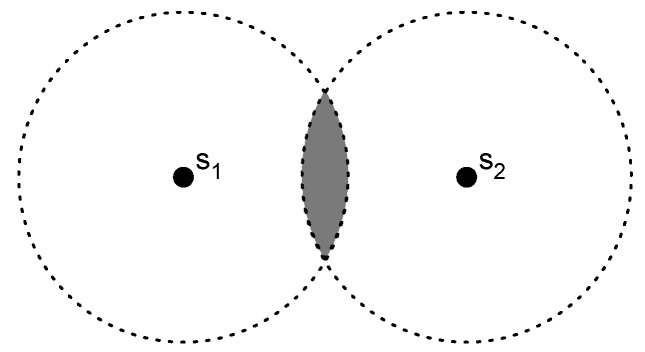
Proximity method concept.

Figure 5 describes the method, where the circles represent the coverage area of the anchor nodes. Any user located in the circle of node $s_{1}$ will estimate its own position as the position of the anchor node $s_{1}$ whereas if the object is located in the circle of anchor $s_{2}$ it will estimate the position as the position of the anchor node $s_{2}$. In the intersection of both circles the selection of the anchor node will be done in terms of the RSS.

One of the first systems to employ the proximity method was the Active Badge system [24]. This system uses a network of infrared sensors that detects the signals transmitted by the active badge and provides a localization estimation with room accuracy. More recently, some authors decided to employ Bluetooth Low Energy (BLE) beacons. For example in [27] a set of BLE beacons are deployed in a hospital for the tracking of the patients. Other works employing BLE technology can be found in [28,29]. It is expected that BLE beacons will be deployed in millions of buildings around the world in a near future. Thus, there will be no need for investment in infrastructure reducing the cost of the system at a minimum as modern smartphones are equipped with BLE transceivers and can be used as positioning devices.

The error committed by the proximity methods is directly related to the size of the coverage areas. Furthermore, if the coverage area of the anchor nodes is reduced, the number of anchor nodes needed for a total coverage of an indoor area increases. For this reason, the Radio-Frequency Identification (RFID) technology is particularly interesting for proximity methods because the deployment of a large number of tags in a building does not escalates the cost of the system [25]. Moreover, the use of passive RFID tags reduces the maintenance cost of the network as the battery of the anchor nodes must not be regularly replaced.

A more general way of using connectivity information is employed in the centroid algorithm, where the estimation of position is computed as the centroid of the position of the anchor nodes received [26], that is,

(4) $$\hat{m}=\left[\frac{1}{N}\sum_{i=1}^{N}x_{i},\frac{1}{N}\sum_{i=1}^{N}y_{i}\right],$$

where $\hat{m}$ is the estimated position of the mobile node and $x_{i}$, $y_{i}$ are respectively the *x* and *y* coordinates of the *i*-th anchor node. In the centroid method the accuracy of the position estimation is also dependent on the number of nodes. In general, proximity based methods cannot obtain highly accurate position estimations, since the obtained accuracy is in the order of the average distance between the anchors deployed in the building. However, the simplicity of these methods offers a good solution for room accuracy systems based on low complex wireless networks.

Examples of proximity methods based on RFID can be found in [73,74,75] where the authors review the RFID based localization methods available in the literature.

Proximity methods provide position estimation with room accuracy using low complex algorithms. The scalability of the system can be achieved by adding more nodes to the network in order to increase the area of coverage. Finally, the cost of the system is low if passive RFID tags are employed as a large number of tags can be deployed at a low price.

#### 2.2.2. Fingerprinting

**Figure 6:**
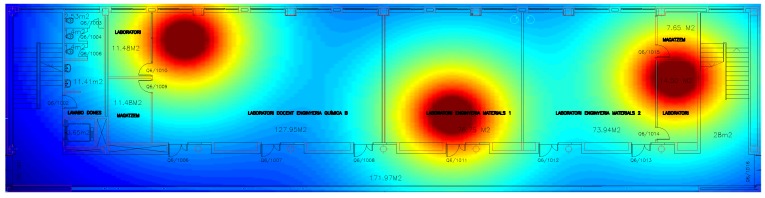
Simulated distribution of RSS in an indoor scenario.

Fingerprinting methods are based on the uniqueness of radio signals received at different positions, which is due to the propagation issues in the complex indoor environment. Usually, in indoor environments different kinds of radio signals can be received , such as the ones received from WiFi, WSN or Bluetooth networks deployed in the buildings among other signals as for example GSM or LTE signals. The complexity of the indoor environment produces big differences between the signals received at different locations due to multipath, shadowing or the propagation in NLOS environments. Figure 6 shows the distribution of the RSS in an indoor environment with three deployed anchor nodes under ideal propagation conditions. The color changes from blue to red as a function of the aggregated received power of the three anchors. The received power generates different subareas that can be easily identified. Note that this effect can be magnified by including the multipath and NLOS to the propagation model considered.

The main idea behind the fingerprinting method is to generate a database of the characteristics of the signals at different positions (fingerprints) and then compare the signals received by the user with the database and estimate the position of the user as the position of the fingerprint that best matches the received signals.

There are two kind of fingerprinting methods: (i) deterministic and (ii) probabilistic. One of the first deterministic fingerprinting system was the RADAR system developed by Microsoft [30]. The system collects the RSS and SNR as fingerprints from a WiFi network and reports an accuracy of 3m. The position is estimated as the position of the fingerprint that minimizes the Euclidian distance between the online measurements and the fingerprints. The search methodology employed is the k Nearest Neighbours (kNN) approach. Similarly, in [31] authors present a fingerprinting method based on the weighted extension of the kNN algorithm. The advantage of the kNN approaches is the reduced computational complexity of these algorithms. There are other systems that increase the accuracy of the position estimation at the expense of a higher computational cost, such as systems based on Support Vector Machines (SVM) [76] or linear discriminant analysis [32].

In the group of the probabilistic approaches the aim is to find the location with maximum likelihood. The Horus system [35] uses a probabilistic model of the signal distribution in the environment and computes the position with maximum posterior probability. There are other systems based on Bayesian networks [36] or on the Kullback-Leibler divergence [37].

The collection of fingerprints is not reduced to the measurement of the characteristics of radio signals, recent works proved that it is possible to use the magnetic field [77]. A study of the feasibility of magnetic fingerprints is performed in [78]. The main advantage of magnetic field fingerprinting methods over RSS fingerprinting methods is that the magnetic field is more stable with time. However, the discernibility of the magnetic field is lower, the same value of the magnetic field can be found in many different parts of the building. Typically, the fingerprinting methods based on the magnetic field compare sequence of fingerprints instead of point to point comparisons. Then, the Dynamic Time Warping (DTW) algorithm is used to identify the correspondence between the online sequence of magnetic fingerprints and the stored database [33]. Similarly, in [34] authors propose the use of the Smith-Waterman algorithm for the alignment of the sequences. Alternatively, the signals transmitted by communications systems like GSM, LTE or Digital Audio Broadcast (DAB) also known as Signals of Opportunity (SOP) can be employed as fingerprints for a positioning system. A comparison of different opportunistic signals for localization can be found in [79]。

The creation of the database samples requires an intensive campaign of measurements in order to collect the fingerprints of the radio signals and create a radio map of the indoor environment. This process is time consuming and vulnerable to environmental changes. Furthermore, the accuracy of the system depends on the assumption of similar wireless conditions between the collection of the fingerprints and the current signals [8]. The movement of humans or objects inside the building will produce differences between the database and the online collected measurements that will cause an increase in the positioning error.

The accuracy of a fingerprinting method is related to the number of points of the calibrated radio map [80]. However, the dimension of the radio map is determined by the application and type of fingerprinting method employed. For example, deterministic methods generally imply less calibration effort than probabilistic methods because the later imply the computation of the statistics of the signals at each calibration point [81]. Similarly, the localization of a person moving around an indoor environment will require a larger calibration campaign than the localization of a static object. Recently researchers have shown an increasing interest in reducing the effort of the calibration process [8]. Besides the typical point to point calibration process that involves a larger calibration effort, the authors proposed different calibration procedures as for example the collection of measurements through a walking path or crowdsourcing between the measurements collected by different users. In [82] authors evaluate the performance of different radio map construction methods. More information about calibration methods for fingerprinting systems can be found in [8].

Independently of the source of fingerprints the main disadvantage of fingerprinting methods is the effort needed for the collection of the database samples, which increases the cost and reduces the scalability.

## 3. Inertial Based Systems

In previous section, we have seen that the network based systems estimate the position of the user measuring the features from the signals received from a wireless network; however, inertial based systems compute their own position without any help from a physical infrastructure. The inertial sensors measure the forces applied to the sensor and thus the movement of the object where the sensor is mounted can be computed. Usually, inertial sensors are mounted together forming Inertial Measurement Units (IMU), which are formed by a 3 axis accelerometer, a 3 axis gyroscope and a 3 axis magnetometer (The magnetometer is not an inertial sensor, however in this work we group it into the inertial measurement unit as this is the typical term used in the literature.). There are two main kinds of inertial navigation systems [6]:

- **Strapdown systems:** these systems integrate twice the acceleration of the user in order to estimate the position.
- **Step and Heading Systems (SHS):** these systems estimate the position by adding to the initial position estimation vectors representing the step length and the step heading of the user.

**Figure 7:**
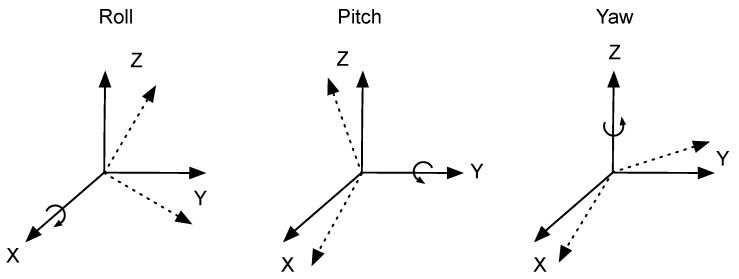
Roll, pitch and yaw angles.

Regardless of the approach used, the first step of an inertial navigation system is the computation of the relative orientation of the sensor and the body of the user. The measurements of an IMU are expressed in the sensor coordinate frame, whenever we attach the IMU to the body of the user, the axes of the sensor coordinate frame may not coincide with the axes of the navigation frame. Any misalignment in the axes produces errors in the measurements; therefore the estimation of the relative orientation is a crucial part of an inertial navigation system. The relative transformation between two coordinate frames can be obtained by sequentially rotating around three axis, where the angles of rotation are expressed as Euler angles, that is, the roll ($\phi_{x}$), pitch ($\theta_{y}$) and yaw ($\psi_{z}$). The definition of the Euler angles is shown in Figure 7.

The transformation between coordinate frames is done using the following rotation matrices [83]:

(5) $$O_{\phi_{x}}=\left[\begin{array}{ccc} 1 & 0 & 0\\ 0 & -\cos(\phi_{x}) & \sin(\phi_{x})\\ 0 & \sin(\phi_{x}) & \cos(\phi_{x}) \end{array}\right],$$

(6) $$O_{\theta_{y}}=\left[\begin{array}{ccc} \cos(\theta_{y}) & 0 & \sin(\theta_{y})\\ 0 & 1 & 0\\ -\sin(\theta_{y}) & 0 & \cos(\theta_{y}) \end{array}\right],$$

(7) $$O_{\psi_{z}}=\left[\begin{array}{ccc} \cos(\psi_{z}) & \sin(\psi_{z}) & 0\\ -\sin(\psi_{z}) & \cos(\psi_{z}) & 0\\ 0 & 0 & 1 \end{array}\right],$$

where O represents the rotation matrix. The rotations are applied in the following order:

(8) $$O_{T}=O_{\phi_{x}}O_{\theta_{y}}O_{\psi_{z}},$$

and the measurements of the IMU in the navigation frame $z_{IMU}^{NF}$ are obtained by multiplying the IMU measurements in the sensor frame $z_{IMU}^{SF}$ by the rotation matrix $O_{T}$, that is,

(9) $$z_{IMU}^{NF}=O_{T}\;z_{IMU}^{SF}.$$

In order to estimate the rotation angles, the earth gravitational field, measured by the accelerometers, is typically employed. In the absence of any external acceleration, the output of an accelerometer corresponds to the earth gravitational field. Therefore it is possible to estimate the roll and pitch angles knowing that if the sensor coordinate frame is aligned with the earth coordinate frame, the gravitation vector must fall in the *z* axis [83], that is,

(10) $$\tan\phi_{x}=\frac{a_{y}}{a_{z}},$$

(11) $$\tan\theta_{y}=\frac{-a_{x}}{\sqrt{a_{y}^{2}+a_{z}^{2}}},$$

where $a_{x}$, $a_{y}$ and $a_{z}$ are the outputs of the accelerometer in the *x*, *y* and *z* axis, respectively. Unfortunately the gravitational field is invariant to the rotation of the yaw angle and therefore the yaw angle remains unknown using this method. This fact is circumvented in indoor positioning systems by assuming knowledge about the initial orientation of the user or by computing the initial orientation using the earth magnetic field.

### 3.1. Strapdown Systems

**Figure 8:**
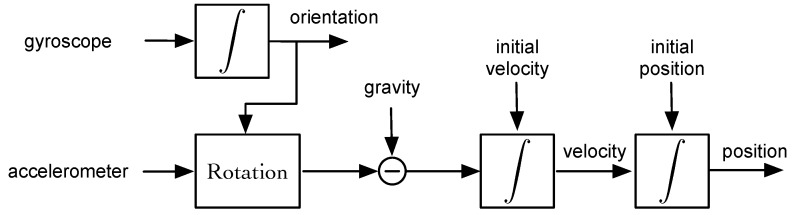
Strapdown navigation system.

Strapdown inertial navigation systems are based on the concept that the position is the double integration of the acceleration. Thus, the first integration of the acceleration signal $a\left(t\right)=\left[\begin{array}{c} a_{x}\left(t\right),a_{y}\left(t\right),a_{z}\left(t\right) \end{array}\right]$ produces the velocity and the integration of the velocity produces the position [84], that is,

(12) $$v\left(t\right)=v\left(0\right)+\int_{0}^{t}\left(a\left(t\right)-g\right)\;dt,$$

(13) $$m\left(t\right)=m\left(0\right)+\int_{0}^{t}v\left(t\right)\;dt,$$

where v is the velocity, g the gravity and m the position, all of them related to the navigation frame. Figure 8 shows the block diagram of a strapdown navigation system. First, the angular velocity measured by the gyroscope is integrated in order to track the orientation of the sensor frame with respect to the navigation frame. Note that once the initial orientation is known, the orientation at any time can be known by accumulating the rotation done in each axis, which is measured by the gyroscope. Once the orientation is known, the signal from the accelerometer is rotated to the navigation frame and the gravitation force is subtracted before the integration of the acceleration signal to obtain the velocity and the position.

The errors in the measurements of the sensors affect differently to the estimation of position. On the one hand, the errors of the accelerometer produce a drift in the position because the integration procedure accumulates the errors over time. On the other hand, the errors of the gyroscope result in an erroneous rotation matrix and therefore the measurements of the accelerometer are incorrectly projected into the navigation frame. Furthermore, the strapdown navigation systems subtract the value of the earth gravitational field before the integration. Any error in the alignment of the frames will produce a bias due to a gravitational component projected to the horizontal plane. This source of error cannot be neglected as the magnitude of the acceleration caused by the gravity is usually greater than the acceleration produced by the movement of the user. In fact, the errors in the gyroscope measurements are the ones limiting the accuracy of the inertial strapdown systems. In general, the error in the estimation of position grows cubically with time due to the integration of the accelerometer and gyroscope signals. Using the current MEMS technology, the estimation of position will deviate over the meter in seconds making the estimation of the trajectory of a human in the long term unfeasible [85].

Recently, Foxlin et al. [38] demonstrated that using a foot mounted IMU the time dependency of the position estimation errors in strapdown systems, which typically grows cubically with time, can be reduced to a linear growth if the Zero Velocity Update (ZUPT) is applied. The idea behind ZUPT is to detect the stance phases of the human walking, when the foot is firmly planted on the ground and the velocity is zero, and apply these zero velocity measurements to an extended Kalman filter that estimates the errors of the inertial measurements. However, the ZUPT strategy cannot correct the errors in the yaw angle. In order to amend this, several authors proposed techniques for reducing the gyroscope bias, such as the zero angular rate update [86] or the heuristic heading reduction [87]. An example of a inertial strapdown system using theses techniques can be found in [39]. Similarly, the authors in [40] applied these updates using an Unscented Kalman Filter (UKF) for the estimation of the inertial measurement errors.

### 3.2. Step and Heading Systems

Contrarily to the strapdown navigation systems, the step and heading systems do not use the integration of the acceleration signal to compute the position of the user. Instead, these systems detect the steps and estimate the length and heading of each step from the accelerometer and gyroscope signals. Then recursively estimate the position of the user by accumulating vectors that represent the movement of the user at each step, that is,

(14) $$m_{x}\left(k\right)=m_{x}\left(k-1\right)+l_{step}\left(k\right)\cos\left(\theta \left(k\right)\right),$$

(15) $$m_{y}\left(k\right)=m_{y}\left(k-1\right)+l_{step}\left(k\right)\sin\left(\theta \left(k\right)\right),$$

where $m_{x}$ and $m_{y}$ are respectively the *x*, *y* components of the position, *k* is the time index, $l_{step}$ the step length and θ the heading. The fundamental cycle for a step and heading system is [6]:

- Identification of the subset of data of an individual step.
- Estimation of the step length.
- Estimation of the heading.

**Figure 9:**
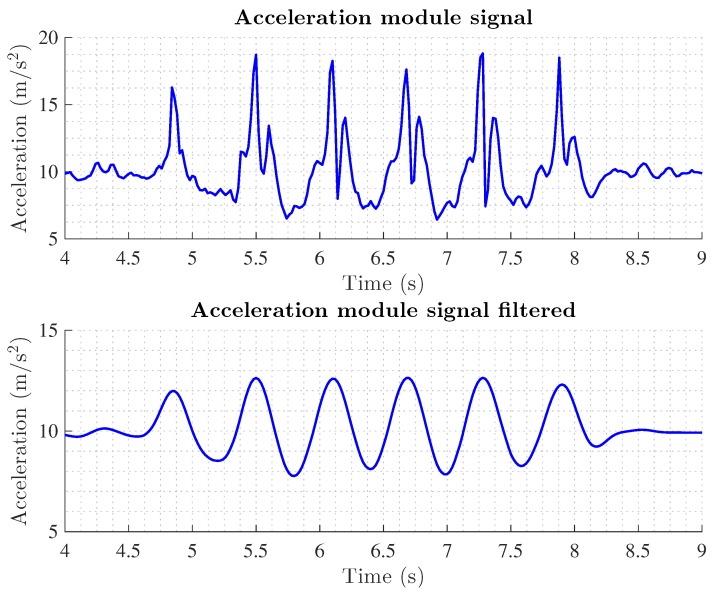
Acceleration signal measured on the hip of a pedestrian during a walk.

Typically, the step of a pedestrian is divided into two phases: (i) the stance phase where the foot is firmly planted on the ground and (ii) the swing phase where the foot is in the air. Most of the algorithms designed to identify step events are based on the detection of the stance phase. Usually, threshold based methods are used to identify the lack of activity measured by the IMU during the stance phase. Traditionally these methods are based on the magnitude of the acceleration but the angular velocity has also been employed [6]. Alternatively there are methods that detect repetitive events on the walking data. Figure 9 shows the module of the acceleration during a walk of a pedestrian, the raw data and the filtered data is shown as many methods filter the data to eliminate high frequencial noise components of the accelerometer measurements. The detection of the steps can be done by counting the number of peaks produced by the strike of the heel in the floor [88]. Other methods compute the zero crossings of the acceleration signal after subtracting the gravity [89]. More complex methods correlate the received signal with a pre-stored template of the acceleration during a step [90]. Due to the repetitive behavior of the acceleration during the steps, spectral analysis is also employed to detect peaks in the typical stepping frequencies [91]. Recently, in [41] authors present a step detection method based on the pitch angle measured by the gyroscope of a smartphone placed in the pocket of the user.

The estimation of the step length can be obtained from the vertical displacement of the pelvis as shown by Weingberg et al. in [92]. Following this procedure the step length is estimated as,

(16) $$l_{step}=K\sqrt[{4}]{a_{z_{max}}-a_{z_{min}}},$$

where *K* is a user-specific constant and $a_{z_{max}}$, $a_{z_{min}}$ are respectively the maximum and minimum of the acceleration in the vertical axis. The step length can also be estimated as a linear function of the step frequency considering that the step length and frequency increase with the speed of the user [91].

Finally, the last point of the fundamental cycle of step and heading systems is the estimation of the heading of each step. The heading estimation of these systems is equal to the strapdown systems, that is, the heading is obtained by the integration of the gyroscope signal. Thus, the final position estimation is drifted by the errors accumulated during the integration. Fortunately, in the step and heading systems the growth of the error is linear with time instead of the cubic error of strapdown systems. The heading can be obtained also using a magnetometer but in indoor environments that include ferromagnetic materials, the accuracy of the heading estimation is degraded. The fusion of both measurements has shown relatively good accuracy in [54] as both measurement errors are complementary, that is, the gyroscope produce high accurate measurements in the short term and the magnetometer gives low accurate measurements but stable in time.

An example of a step and heading system is found in [42], where the authors design a system for hand held smartphones. Similarly, in [41] a SHS for smartphones placed in the pocket of the user is designed where the steps are detected using the gyroscope signal. A comparison of the performance of different systems using low cost sensors is presented in [43].

Despite the improvements of the SHS in the reduction of the drift, it is still present and therefore these kind of systems cannot be applied for a long period of time without any correcting strategy.

Inertial based systems are completely scalable in terms of size and number of users as there is no need of deploying infrastructure in the environment. Furthermore, nowadays smartphones already include an embedded IMU and thus the cost of the system is minimum. However, as has been said, it is important to take into account the accuracy reduction produced by the inertial drift.

### 3.3. Simultaneous Localization and Mapping

The Simultaneous Localization and Mapping (SLAM) extends the localization problem including the estimation of a map. It was developed by the robotics community and the key idea is that a mobile robot moving in and indoor environment can build a consistent map of the building at the same time that determines its own position without prior information about the position or the map [93,94].

**Figure 10:**
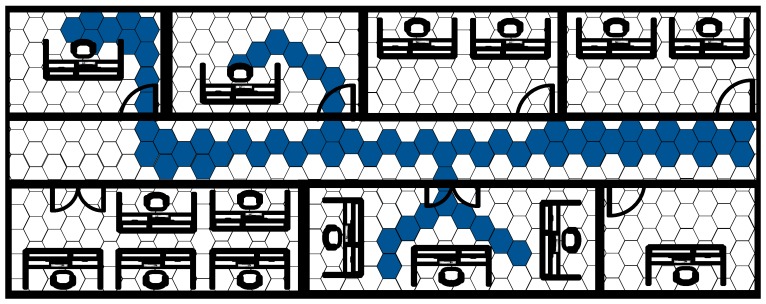
Simultaneous localization and mapping.

In 2012, Angermann et al. developed the FootSLAM system resulting from the application of the SLAM problem to the localization of a pedestrian in an indoor environment [44]. The FootSLAM system maps the environment with a regular grid of hexagons and builds a probabilistic map computing the probability that a pedestrian crosses the transition between two adjacent hexagons. The idea beyond this system is that it is probable that a pedestrian walking in an indoor environment passes different times by the same place and thus the estimation can be enhanced considering that the user goes and returns along the same path. Figure 10 shows the concept of the FootSLAM where the more likely hexagons are highlighted. The FootSLAM system computes the odometry of the user using a foot mounted IMU and uses the movement of the user between two epochs to update the particles of a Rao-Blackwellized particle filter. Each particle takes into account a possible path of the user and computes the corresponding hexagonal probabilistic map. At every epoch, the estimated path and probabilistic map of each particle are updated with the measured movement of the user, that is, the probabilities of the transitions between hexagons crossed due to the movement of the user are increased. Thus, whenever the user closes the loop and returns to the origin the filter will reward those particles that have gone and returned along the same path. With this method the drift of the inertial sensors can be eliminated but the filter has the dependence on the closure of the loops. If the walk of the pedestrian does not return to the same place the error in the position estimations will grow as in the typical step and heading systems. The main disadvantage of the system is the computational complexity as every particle must store a probabilistic map of all the environment which can lead to high computational complexity for large environments.

Recently, there appeared works in the literature based on the FootSLAM system, such as the FeetSLAM where the maps of different users are combined [45] or the PocketSLAM where the inertial measurements are obtained from an smartphone placed on the pocket of the user [46].

Although SLAM approaches obtain highly accurate position estimations when the user walks in loops and the cost of the systems is reduced to the cost of the IMU, the computational complexity grows with the size of the map and therefore the scalability of the system is limited by the area of the environment.

## 4. Hybrid Positioning Systems

An hybrid positioning system by definition is a system that combines two or more systems in order to enhance the performance offered by these systems individually. Currently, there are myriads of hybrid positioning systems in the literature that combine the different IPS reviewed so far.

A complete classification of hybrid positioning systems is not feasible due to the large amount of possible combinations of IPS that can form an hybrid system. Therefore, in this section we will only review the most interesting systems for mass market applications. Table 2 and Table 1 present a comparison between the IPS methods reviewed in the previous sections including the main characteristics of each method. Furthermore, the main problems in the scalability of the systems are included. For example, angle and time based methods require additional hardware and therefore the cost of the system increases. Moreover they cannot be applied to a wireless network if they are not synchronized with the nodes of the network. Therefore, those systems are not the best option for mass market applications. Similarly, fingerprinting methods require a laborious calibration campaign for every indoor environment and thus they are also not valid for mass market applications. The rest of the systems, that is, RSS, proximity, PDR and SLAM, are valid for mass market applications but the combination of the RSS with the PDR is the most interesting one as it provides accurate results with low monetary cost and low/medium computational cost. In particular, we will focus on the following three groups:

- **RSS-IMU hybrid systems:** here we include the methods that combine inertial measurements with RSS measurements either by using a propagation model or a fingerprinting approach.
- **Map hybrid systems:** here we embrace the methods that in addition to the RSS and/or IMU measurements also use the map of the building to enhance the performance of an IPS.
- **Smartphone hybrid systems:** here we include those RSS-IMU and Map hybrid systems that have been specifically designed for smartphones.

### 4.1. RSS-IMU Hybrid Systems

The availability of wireless networks deployed inside millions of buildings around the world make RSS based positioning systems an attractive option for mass market hybrid systems because there is no need of investing in a wireless infrastructure. Note that, as stated in Section 2.1.3 the RSS can be computed just listening to the network, i.e., without any additional hardware, as far as most of the wireless standards of communication already include the RSS field in the radio packets.

The most common kind of RSS hybrid system is one that combines it with inertial sensors. The motivation is clear: both systems have complementary errors. The inertial based systems obtain highly accurate positions estimations in the short term while the RSS based systems are less accurate but the estimations of position are time invariant. An example of this kind of hybrid systems is found in [47] where the authors developed a system that combines the position estimation of a WiFi probabilistic fingerprinting with the information of a foot mounted SHS using an Extended Kalman Filter (EKF) for the fusion of the systems. Similarly, in [48,49,64] the step information of a hip mounted IMU is combined with the position estimations of a range based RSS system. Jimenez et al. [50] combine a strapdown foot mounted inertial system with the RSS of RFID tags using an EKF. Table 3 summarizes the RSS-IMU hybrid positioning systems showing the main characteristics of the underlying RSS and IMU systems as well as the parameters and results of the experimental evaluation.

### 4.2. Map Hybrid Systems

**Figure 11:**
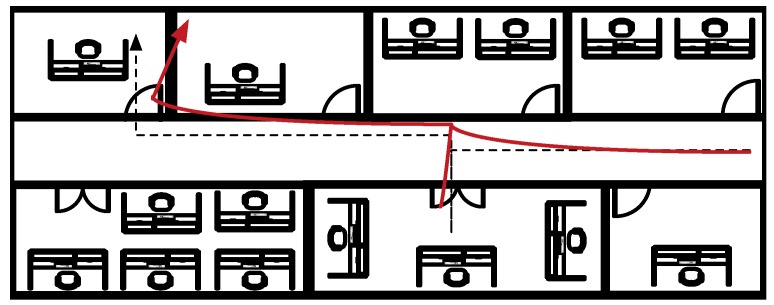
Inertial position estimation with drift (red) and corrected path (dashed).

The high complexity of the indoor environments with different distributions of walls and furniture that produces NLOS communications between the user and the wireless networks is an inconvenient for IPS because it produces less accurate estimations of the position. However, if the map of the building is a priori known by the user, the high complexity can be an advantage to the IPS as it can constrain the possible positions and improve the accuracy of the estimations. Commonly, the map information is used to enhance the performance of the RSS-IMU hybrid systems. Figure 11 shows the estimated trajectory from an inertial system that is affected by drift and how the map information can help us to recover the original path. Typically, the map information is included in the fusion of the measurements using a particle filter. During the calculation of the weights of each particle, the map constraints are calculated and those particles that have been propagated to impossible locations (as for example crossing a wall) receive a weight of zero preventing the resampling of this particle in the following epoch. For example, in [51] the measurements of an RSS probabilistic fingerprinting are combined with the measurements of a belt mounted SHS. Then a PF fuses the measurements with the map information. Similarly, in [52] the authors use an equivalent system but the IMU is placed on the foot of the user. There are other examples of hybrid systems with map information as in [53,54]. Table 4 summarizes all of them for the purpose of comparison including the main characteristics of the underlaying systems employed and the experimental evaluation.

### 4.3. Smartphone Hybrid Systems

The popularization of smartphones among the world converted the smartphone in the perfect device for positioning. Any IPS that can be implemented in a smartphone has the potential to be used by millions of people, granting access to the mass market without the need of investing in devices for positioning. For this reason the research efforts of authors working in the field of pedestrian positioning focused on the smartphone technology during the last years. Furthermore, the different technologies included in the nowadays smartphones allow to implement hybrid systems using a single device. Note that a smartphone usually includes WiFi, GSM, LTE and Bluetooth radios as well as an IMU among other technologies like GPS.

Many different examples of IPS based on smartphones can be found in the literature. For example in [55] an IPS for underground public transport systems is developed based on the information about the routes and inertial sensors. Another example can be found in [56], where the authors use a Least Square Support Vector Machine (LS-SVM) for the classification of the smartphone position (hand, pocket, head, etc.) and then combine the inertial data with the measurements from a WiFi fingerprinting method using a Hidden Markov Model (HMM). Notwithstanding, typically the position of the smartphone is fixed by the designer of the system. In [63] the authors combine the inertial measurements with RSS and magnetic fingerprinting using an EKF. Other authors employ the SLAM approach in the smartphone combining the inertial measurements with WiFi and magnetic fingerprints [57,58]. In [65] authors combine inertial sensors with a WiFi fingerprinting method and employ BLE beacons for the correction of the inertial drift. Similarly, in [66] authors combine inertial sensors with BLE and WiFi measurements employing and Extended Kalman Filter. More accurate results can be obtained if the map information is available. In [59] the authors also use a HMM for the fusion of the WiFi and inertial measurements and incorporate the map information. Similarly, [60,61] combine the WiFi fingerprints with the inertial measurements using a particle filter and in [62] the fusion is done with a Kalman filter. Authors in [67] combine the inertial measurements of a smartphone with the RSS measurements of a smartphone and a smartwatch through an EKF. The performance of these systems is summarized in Table 5.

## 5. Conclusions

This work has surveyed the Indoor Positioning Systems (IPS) with a special interest in those IPS that can be applied to mass market applications. First a general classification of IPS is performed and each group is reviewed analysing the advantages and disadvantages of each system in terms of accuracy, cost and scalability. Then the hybrid systems are reviewed focusing on the current solutions available in the literature that can be applied to mass market applications. Finally, we also reviewed the state of the art of IPS based on smartphones because the popularization of the smartphones around the world is a key advantage for reducing the cost of the implementation of an IPS for mass market applications.

Considering the special requirements of mass market applications, that is, accuracy, cost and scalability, we can conclude that over the large amount of IPS available in the literature, the most interesting ones for mast market applications are the RSS-IMU based hybrid systems combined with map information whenever it is available. These systems, can provide accuracies in the order of 1 meter in large indoor scenarios. Furthermore, the cost of the system is reduced if the WiFi network already deployed in lots of buildings is employed for positioning or low cost technologies are employed as RFID or BLE beacons. The scalability of the system can be guaranteed if range based or proximity methods are employed because these methods require little or no calibration effort. Note that these systems can be implemented with nowadays commercial smartphones and that in the case of employing smartphones different wireless technologies can be employed (WiFI, BLE, ...) increasing the positioning accuracy without increasing the cost of the system.

Although there have been great advantages in the research of IPS and the current technology is increasing the positioning accuracy continuously, the positioning problem in indoor environment is far from being solved and more research is needed in order to reach the positioning accuracy demanded by indoor location based services. From the timely and comprehensive review of the literature, this survey may further encourage new research efforts and for this reason we briefly suggest here some future research lines. In particular, we focus on the open research problems of IPS based on smartphones as we consider the smartphone as the most promising device for indoor positioning systems applied to mass market applications.

*Adaptability to smartphone placement*: Smartphones are devices employed for many different applications that involve many different positions of the smartphone with respect to the body of the user. Typically a smartphone can be held in the pocket, in the hand, next to the head, in a bag or purse among many other possible positions. New IPS should take into account the position of the smartphone in order to compute the relative orientation of the inertial sensors embedded in the smartphone with respect to the navigation frame.

*Heterogeneity of transceivers*: In a mass market application based on smartphones, each user will employ its own smartphone as a positioning device. With the large amount of different smartphone available in the market, we encounter devices with different sensitivities, different antenna patterns, etc. Thus, different smartphones will measure different RSS in the same conditions and this fact should be taken into account in the design of new IPS.

*Battery life*: It is well known that battery lifetime in many commercial smartphones is not as good as desired. The use of fusion algorithms like the Kalman filter or the Particle filter increases the computational complexity of the IPS and reduce the battery life of the smartphones. Therefore, it is crucial to develop new energy efficient algorithms with reduced computational complexity which do not drain the battery of the smartphones.

*Smartwatches*: As it happened with the smartphones years ago, the popularity of smartwatches is increasing every day. Most of them already include inertial sensors and WiFi and Bluetooth transceivers so we encourage the development of IPS based on multiple devices.

## Figures and Tables

**Table 1:** Overview of Indoor Positioning Systems.

System	Type	Cost	Scalability	Anchors	Area ($m^{2}$)	Error	
						Type	Value
Harter et al. [12]	Time	Expensive	Limited	100	280	95th	0.09 m
Priyantha et al. [13,95]	Time	Medium	Limited	6	<10	90th	0.3 m
Correa et al. [22]	RSS	Low	Yes	6	530	RMSE	1.4 m
Palumbo et al. [23]	RSS	Low	Yes	8	36	75th	1.8 m
Yang et al. [19]	RSS	Low	Yes	5	3400	median	3 m
Lin et al. [27]	Proximity	Low	Yes	12	300	room detection	97.2 %
Bolic et al. [25]	Proximity	Low	Yes	24	8	RMSE	0.32 m
Bahl et al. [30]	Fingerprinting	Medium	Limited	3	980	75th	4.69 m
Han et al. [31]	Fingerprinting	Medium	Limited	3400	192,200	75th	3–9 m
Youssef et al. [35]	Fingerprinting	Medium	Limited	21	1700	90th	1.4 m
Wu et al. [34]	Magnetic fingerprinting	Low	Limited	0	4000	90th	2.5 m
Foxlin et al. [38]	Inertial	Low	Yes	0	75	% travelled path	0.3 %
Jimenez et al. [39]	Inertial	Low	Yes	0	3600	% travelled path	0.3–1.5 %
Angermann et al. [44]	Inertial	Low	Limited	0	600	RMSE	1–2 m

**Table 2:** Comparison of Indoor Positioning Systems.

System	Accuracy	Drift	Cost		Calibration	Integration with Network	Hardware	Scalability problems
			Computational	Monetary				
Time	High	No	Low	Medium	No	Yes	Transceiver, accurate clocks	Synchronisation
Angle	High	No	Low	Medium	No	Yes	Transceiver, multiple antennas	Synchronisation
RSS	Low	No	Low	Low	Easy	No	Transceiver	No
Proximity	Poor	No	Low	Low	No	No	Transceiver	No
Fingerprinting	Medium	No	High	Low	Laborious	No	Transceiver	Calibration
PDR	High	Yes	Medium	Low	No	No	IMU	No
SLAM	High	Closed loops	High	Low	No	No	IMU	No

**Table 3:** RSS-IMU hybrid positioning systems.

System	Technologies	RSS	IMU		Anchors	Area ($m^{2}$)	Error (m)	Cost	Scalability
			Position	Method					
Frank et al. [47]	WiFi, MEMS	Fingerprinting	Foot	SHS	11	Floor	1.65	Medium	Limited by calibration
Schmid et al. [48]	WSN, MEMS	Propagation model	Hip	SHS	62	1125	4	Low	Yes
Tarrío et al. [49]	WSN, MEMS	Propagation model	Waist	SHS	9	100	2.3	Low	Yes
Correa et al. [64]	WSN, MEMS	Propagation model	Waist	SHS	8	155	0.9	Low	Yes
Jiménez et al. [50]	RFID, MEMS	Propagation model	Foot	Strapdown	71	2200	1.35	Low	Yes

**Table 4:** Map hybrid positioning systems.

System	Technologies	RSS	IMU		Anchors	Area ($m^{2}$)	Error		Cost	Scalability
			Position	Method			Type	Value (m)		
Evennou et al. [51]	WiFi, MEMS	Fingerprinting	Belt	SHS	4	1600	RMSE	1.53	Medium	Limited by calibration
Woodman et al. [52]	WiFi, MEMS	Fingerprinting	Foot	SHS	33	8725	90th	0.73	Medium	Limited by calibration
Wang et al. [53]	WiFi, MEMS	Fingerprinting	N/A	Step	5	1000	RMSE	4.3	Medium	Limited by calibration
Klingbeil et al. [54]	WSN, MEMS	Proximity	Belt	SHS	9	Floor	RMSE	1.2	Low	Yes

**Table 5:** Smartphone positioning systems.

System	Technologies				Fusion Method	Area ($m^{2}$)	Error		Cost	Scalability
	WiFi	IMU	Magnetic	Map			Type	Value (m)		
Pei et al. [56]	Yes	Yes	No	No	HMM	Building	RMSE	4.55	Medium	Limited by calibration
Faragher et al. [57]	Yes	Yes	Yes	Yes	SLAM	450	95th	2.7	Medium	Limited by calibration and complexity
Liu et al. [59]	Yes	Yes	No	Yes	HMM	Floor	RMSE	3.1	Medium	Limited by calibration
Radu et al. [60]	Yes	Yes	No	Yes	PF	Floor	90th	6	Medium	Limited by calibration
Moder et al. [61]	Yes	Yes	No	Yes	PF	Building	90th	2.3	Medium	Limited by calibration
Chen et al. [62]	Yes	Yes	No	Yes	KF	3800	RMSE	1	Medium	Limited by calibration
Li et al. [63]	Yes	Yes	Yes	No	EKF	8400	RMSE	2.9	Medium	Limited by calibration
Correa et al. [67]	Yes	Yes	No	No	EKF	6000	RMSE	1.4–3.4	Low	Yes
Zou et al. [65]	Yes	Yes	No	No	PF	600	Mean	0.6	Medium	Limited by calibration
Chen et al. [66]	Yes	Yes	No	Yes	KF	425	RMSE	1.28	Low	Yes
